# The macroeconomic burden of noncommunicable diseases and mental health conditions in South America

**DOI:** 10.1371/journal.pone.0293144

**Published:** 2023-10-20

**Authors:** Maddalena Ferranna, Daniel Cadarette, Simiao Chen, Parastou Ghazi, Faith Ross, Leo Zucker, David E. Bloom

**Affiliations:** 1 Department of Pharmaceutical and Health Economics, Alfred E. Mann School of Pharmacy and Pharmaceutical Sciences, University of Southern California, Los Angeles, United States of America; 2 Schaeffer Center for Health Policy and Economics, University of Southern California, Los Angeles, United States of America; 3 Harvard Kennedy School, Cambridge, Massachusetts, United States of America; 4 Heidelberg Institute of Global Health, Faculty of Medicine and University Hospital, Heidelberg University, Heidelberg, Germany; 5 Department of Global Health and Population, Harvard T.H. Chan School of Public Health, Boston, Massachusetts, United States of America; Facultad Latinoamericana de Ciencias Sociales Mexico, MEXICO

## Abstract

Noncommunicable diseases and mental health conditions (referred to collectively as NMHs) are the greatest cause of preventable death, illness, and disability in South America and negatively affect countries’ economic performance through their detrimental impacts on labor supply and capital investments. Sound, evidence-based policy-making requires a deep understanding of the macroeconomic costs of NMHs and of their distribution across countries and diseases. The paper estimates and projects the macroeconomic burden of NMHs over the period 2020–2050 in 10 South American countries. We estimate the impact of NMHs on gross domestic product (GDP) through a human capital-augmented production function approach, accounting for mortality and morbidity effects of NMHs on labor supply, for the impact of treatment costs on physical capital accumulation, and for variations in human capital by age. Our central estimates suggest that the overall burden of NMHs in these countries amounts to $7.3 trillion (2022 international $, 3% discount rate, 95% confidence interval: $6.8–$7.8 trillion). Overall, the macroeconomic burden of NMHs is around 4% of total GDP over 2020–2050, with little variation across countries (from 3.2% in Peru to 4.5% in Brazil). In other words, without NMHs, annual GDP over 2020–2050 would be about 4% larger. In most countries, the largest macroeconomic burden is associated with cancers. Results from the paper point to a significant macroeconomic burden of NMHs in South America and provide a strong justification for investment in NMH prevention, early detection, treatment, and formal and informal care.

## Introduction

Noncommunicable diseases (including cardiovascular diseases, diabetes, cancers, and chronic respiratory diseases) and mental health conditions, referred to collectively as NMHs, are the greatest cause of preventable illness, disability, and death worldwide. In South America, NMHs cause 77% of all deaths and 72% of all healthy life years lost as measured in disability-adjusted life years (DALYs) by the Global Burden of Disease Study 2019 (GBD) [1]. Cardiovascular diseases and cancers cause the largest mortality and morbidity burdens, and mental health conditions contribute to about 12% of all healthy life years lost in the region (Table A1 in S1 Appendix). Also noteworthy is that about 28% of NMH-related deaths occur prematurely among individuals aged 25–65, when they are considered to be economically more productive.

NMHs, and ill health in general, tend to suppress economic growth [2, 3]. We can expect a negative impact of NMHs on a country’s economic outlook for two main reasons. First, premature death and disability prevent individuals from participating in productive market activities, thereby reducing labor supply. The negative impact of morbidity may be due to early retirement [4, 5], reduced working hours [6], or reduced productivity [7, 8]. Second, unhealthy populations tend to have lower rates of savings, investment, and physical capital accumulation because they spend more resources on healthcare [9, 10]. If the burden of NMHs were reduced, some of those resources could be redirected to more productive investments, e.g., in education or infrastructure. Both channels hamper economic growth and have compound effects because reductions in aggregate income further reduce savings and investments, thereby reinforcing the decline in economic growth.

Despite the large body of literature on the causal impact of health on economic growth [11], no study has yet provided comprehensive estimates of the macroeconomic effects of NMHs in the South American region. The paper aims to fill this gap. Using a human capital-augmented production function approach [12], we estimate and project the impact of NMHs on gross domestic product (GDP) in 10 South American countries: Argentina, Bolivia, Brazil, Chile, Colombia, Ecuador, Paraguay, Peru, Uruguay, and Venezuela. In particular, we simulate through a mathematical model the expected GDP in the 10 South American countries over the period 2020–2050 if all NMHs were eliminated and compare the results with current GDP projections over the same time period. The estimated difference in GDP across the two scenarios represents the macroeconomic burden of NMHs. Our GDP estimates in the absence of NMHs account for mortality and morbidity effects of NMHs on labor supply, for the impact of treatment costs on physical capital accumulation, and for variations in human capital by age.

NMHs cause a significant macroeconomic burden in the South American region. Our central estimates suggest that the overall burden of NMHs in the region amounts to $7.3 trillion (2022 international $) over 2020–2050 (with a 3% discount rate), or 4% of total GDP, with moderate variation across countries. In other words, without NMHs, annual GDP over 2020–2050 would be about 4% larger in all countries.

The literature on the macroeconomic effects of NMHs is scant. One set of papers uses a production function approach similar to the one adopted in this paper [12–19]. Only one study focuses on Latin America and the Caribbean [13], and it estimates that total economic losses (in terms of lost output) associated with NMHs range from $18.45 billion in Jamaica (in 2015 US$) to $81.96 billion in Costa Rica and $477.33 billion in Peru over the period 2015–2030. Compared with [13], we extend the analysis to more countries in the region and employ an enhanced modeling approach that accounts for variations in human capital by age. Human capital accumulation is a key determinant of economic growth [20, 21], but differences in human capital levels across age groups are stark due to age differences in work experience (typically increasing with age) and cohort differences in schooling (with older birth cohorts typically displaying lower levels of schooling). Because NMH prevalence increases with age, accounting for the different levels of human capital across age groups is important to provide a more accurate estimate of the macroeconomic burden of NMHs.

Another strand of literature estimates the macroeconomic effects of NMHs through economic growth regression models (e.g., [22–25]). Compared with our approach, empirical economic growth regression models allow a direct estimation of the overall effect of ill health on growth, net of any economic adjustment mechanisms. However, they are very data-intensive, and generating unbiased estimates is difficult due to common issues such as reverse causality, omitted variables, and measurement errors [26]. In addition, regression models deliver meaningful results only for severe diseases that affect many people (e.g., all NMHs). Detecting significant effects is more difficult for less severe diseases due to small sample sizes [27].

Finally, note that while we focus on the macroeconomic burden of NMHs, other metrics of economic burden exist. Two common approaches are the cost-of-illness (COI) approach (e.g., [28–32]), and the value-per-statistical-life (VSL) approach (e.g., [33, 34]). The COI approach sums all the direct costs (e.g., medical costs and travel costs) and indirect costs (e.g., the value of lost income because of reduced working time) associated with a health condition. The VSL approach infers the burden of a condition from studies on individuals’ willingness to pay to reduce fatal (and nonfatal) risks. Thus, in principle, the VSL approach incorporates both the intrinsic and the instrumental values of good health. In the paper, we compare our measure of macroeconomic burden with the cost of illness attributable to NMHs and with the result of a VSL study. We find that the macroeconomic approach leads to conservative estimates of economic burden compared to both the COI and the VSL approaches. Nevertheless, we find that the economic burden of NMHs in South America is substantial and that it calls for increased investment in NMH prevention and treatment.

## Methods

### Model description

**Fig 1:**
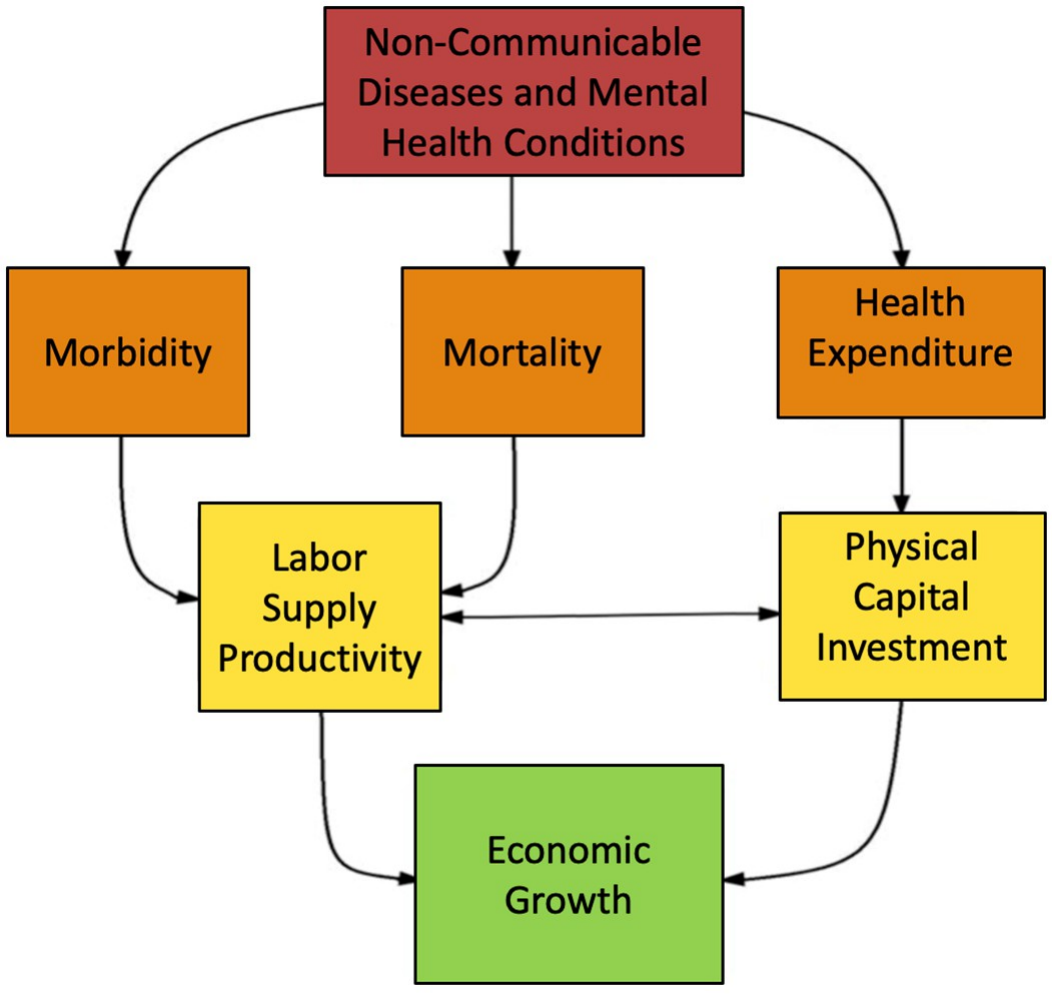
Diagram of the macroeconomic model.

We investigate the macroeconomic burden of NMHs using the human capital-enhanced production function approach developed by [12]. Fig 1 represents the model schematically. Model equations are in S1 Appendix.

Briefly, annual GDP is modeled as a function of effective labor supply and physical capital stock. Effective labor supply accounts for the number of individuals participating in the labor force and for their level of human capital [35]. Both the size of the labor force and the level of human capital depend on the age structure of the population. Following the Mincer model, human capital positively depends on individuals’ educational attainment and their work experience [36]. Thus, countries with a more educated labor force tend to have larger GDP. For a given level of education, older individuals have more work experience and thus more human capital.

Health enters the model in three ways. First, NMHs affect mortality patterns over time and therefore the number of working-age individuals. Second, NMHs cause disabilities that further affect the labor force participation of individuals. Thus, reductions in NMH prevalence positively affect the size of the effective labor force and thus increase GDP. Third, NMHs negatively affect physical capital accumulation because savings may finance part of the treatment costs. Reduction of NMH prevalence saves healthcare resources that can be invested in physical capital. Note that the impact of NMHs on the labor force accounts for decreasing labor force participation as individuals age and for differences in human capital by age.

To estimate the macroeconomic cost of NMHs, we simulate GDP for each country over three decades (2020–2050) in two scenarios: a status quo scenario and a counterfactual scenario. In the status quo scenario, no intervention is implemented. Prevalence of NMHs evolves as expected, and we rely on GDP projections from the International Monetary Fund. In the counterfactual scenario, we assume complete elimination of NMHs at zero cost. The economic burden of NMHs is defined as the (discounted) cumulative difference in GDP across the two scenarios.

In the analysis, we estimate the economic benefits of eliminating all NMHs and the benefits associated with elimination of specific diseases. We focus on the most relevant diseases, including cancers, cardiovascular diseases, chronic respiratory diseases, diabetes and kidney diseases, and mental health conditions. Table 1 presents details on the diseases included in each category.

**Table 1:** List of noncommunicable diseases.

Category^i^	Definition
Noncommunicable diseases	Cancers, cardiovascular diseases, chronic respiratory diseases, diabetes and kidney diseases, digestive diseases, mental disorders, musculoskeletal disorders, neurological disorders, sense organ diseases, skin and subcutaneous diseases, substance use disorders, other benign neoplasms, other NCDs.
Cancers	Bladder cancer, brain and central nervous system cancer, breast cancer, cervical cancer, colon and rectum cancer, esophageal cancer, gallbladder and biliary tract cancer, Hodgkin lymphoma, kidney cancer, larynx cancer, leukemia, lip and oral cavity cancer, liver cancer, malignant skin melanoma, mesothelioma, multiple myeloma, nasopharynx cancer, non-Hodgkin lymphoma, non-melanoma skin cancer, ovarian cancer, other malignant neoplasms, pancreatic cancer, pharynx cancer, prostate cancer, stomach cancer, testicular cancer, thyroid cancer, tracheal bronchus and lung cancer, uterine cancer.
Cardiovascular diseases	Aortic aneurysm, atrial fibrillation and flutter, cardiomyopathy and myocarditis, endocarditis, hypertensive heart disease, ischemic heart disease, non-rheumatic valvular heart disease, other cardiovascular and circulatory diseases, peripheral artery disease, rheumatic heart disease, stroke.
Chronic respiratory diseases	Asthma, chronic obstructive pulmonary disease, interstitial lung disease and pulmonary sarcoidosis, pneumoconiosis.
Diabetes and kidney diseases	Acute glomerulonephritis, chronic kidney diseases, diabetes mellitus.
Mental health conditions	Alcohol use disorders, Alzheimer’s disease and other dementias, anxiety disorders, attention deficit/hyperactivity disorders, autism spectrum disorders, bipolar disorders, conduct disorders, depressive disorders, drug use disorders, eating disorders, idiopathic developmental intellectual disability, headache disorders, idiopathic epilepsy, motor neuron disease, multiple sclerosis, other mental disorders, other neurological disorders, Parkinson’s disease, schizophrenia.

^**i**^**.** The categories follow the classification employed by the Global Burden of Disease 2019.

### Data

Details on the model calibration are included in S1 Appendix. Here we describe the main assumptions we introduce in the model.

We use age- and gender-specific population projections from 2020 to 2050 from the United Nations database [37]. In the simulations, we divide the population aged 0–70 years into 14 five-year age groups and consider individuals aged 70 and older separately. We also distinguish projections by gender.

For each country, GDP projections in the status quo scenario are derived by combining data on purchasing power parity GDP from the World Bank and projected real GDP growth rates from the International Monetary Fund. For Venezuela, we rely on purchasing power parity GDP data from the Central Intelligence Agency (most recent year is 2018).

Projections of age- and gender-specific labor force participation rates are based on data from the International Labour Organization [38]. We rely on the Barro-Lee database for country-, gender-, and age-specific data on average years of schooling [39].

Data on the physical capital stock at the beginning of the simulation are from Penn World Table (the latest available values are from 2019) [40], while status quo projections of annual saving rates are based on gross savings (as % of GDP) in the years 2010–2021 from the World Bank [41].

Absent country-specific data on the impact of having a disability on labor force participation and productivity, the model assumes that mortality and morbidity contribute to labor supply reductions in the same proportions as mortality and morbidity contribute to the health burden of NMHs in terms of DALYs, where mortality is measured in years of life lost and morbidity in years lived with a disability [12]. Projections of disease-specific mortality and morbidity rates are based on data from GBD [1].

To estimate country-level treatment costs, we assume that the per case treatment cost for a specific disease is proportional to the per capita health expenditure in a country, as previous studies have assumed [14, 42]. We estimate this proportion using cost data for the United States from Dieleman et al. [43]. We then derive country- and disease-specific per capita treatment costs by multiplying the estimated proportion by country-specific data on disease prevalence and per capita health expenditures. To account for rising medical costs, we assume that treatment costs will increase over time. We project future health expenditures (% of GDP) using World Bank data for the years 2000–2019 [44] and assuming that they will grow at a constant yearly rate in the future.

### Sensitivity analyses

Considerable uncertainty surrounds the projected health and economic variables. We capture this uncertainty through a probabilistic sensitivity analysis by varying the age- and gender-specific growth rates of mortality and morbidity, the age- and gender-specific growth rates of labor force participation, the GDP growth rate, and the saving rate. We assume that each of these variables is normally distributed, with standard deviations derived from the previously mentioned data. We assume that the parameter distributions are independent and take a Latin hypercube sample of the parameter space with 1,000 draws. We also test the robustness of our results to changes in model assumptions, including the underlying disease burden, the impact of treatment costs on physical capital accumulation, the impact of morbidity on labor force participation, and the discount rate.

Finally, we compare our macroeconomic costs of NMHs with the costs derived from a COI study and from a VSL calculation. The cost-of-illness approach views the burden of NMHs as the sum of several categories of direct and indirect costs, including: personal medical care costs; non-medical costs, such as the costs of transportation for treatment and care; non-personal costs, such as those associated with outreach activities; and personal income losses [30]. Due to data availability, we include only treatment costs and personal income losses in the COI study. We proxy personal income losses from one year of absence from the labor force with annual per capita GDP. The VSL approach reflects the population willingness to pay to reduce the risk of death or disability associated with NMHs. Absent country-specific estimates of such a willingness-to-pay, we follow the conventional approach of assuming that the value of preventing a disability-adjusted year of life lost equals one to five times GDP per capita [45].

## Results

Table 2 shows the macroeconomic burden of NMHs over the period 2020–2050 for each country in the baseline scenario. The baseline scenario assumes a 3% discount rate for GDP and a disease burden based on the mean estimates provided by GBD [1]. The first two columns of Table 2 summarize the *undiscounted* number of DALYs averted over the period 2020–2050 if NMHs were completely eliminated (total number of DALYs and number of DALYs per 1,000 people). The other columns show the economic burden of NMHs using different measures of economic performance: total GDP loss in monetary terms (2022 international $), per capita GDP loss (relative to the mean population over the period 2020–2050), and loss as a percentage of (discounted) total GDP over the period 2020–2050. The last measure was computed by dividing the macroeconomic burden due to NMHs by the discounted sum of annual GDP in the status quo scenario. The table presents the mean estimate of the burden of NMHs; 95% confidence intervals (CIs) are in parentheses.

**Table 2:** Macroeconomic burden of NMHs over the period 2020–2050.

Country	Number of DALYs (millions)^i^	Number of DALYs per 1,000 people^ii^	Total GDP loss (in billions, 2022 international $)	Per capita GDP loss (2022 international $)^iii^	Percentage of total GDP during 2020–2050^iv^
**Argentina**	367 (363–371)	243 (240–246)	1,162 (1,071–1,253)	23,900 (22,000–25,700)	4.4 (4.0–4.7)
**Bolivia**	86 (85–87)	192 (191–192)	109 (95–123)	7,600 (6,600–8,500)	4.0 (3.5–4.5)
**Brazil**	1,622 (1,613–1,630)	232 (231–233)	3,702 (3,472–3,932)	16,400 (15,400–17,400)	4.5 (4.2–4.7)
**Chile**	147 (146–148)	229 (227–232)	566 (536–595)	27,300 (25,900–28,800)	4.4 (4.1–4.6)
**Colombia**	329 (324–334)	193 (190–196)	849 (804–894)	15,500 (14,600–16,300)	3.9 (3.7–4.1)
**Ecuador**	124 (122–126)	197 (194–201)	192 (187–197)	9,500 (9,300–9,800)	3.7 (3.6–3.7)
**Paraguay**	49 (48–50)	205 (203–208)	110 (107–114)	14,300 (13,900–14,800)	4.3 (4.2–4.5)
**Peru**	174 (172–176)	147 (146–149)	376 (362–389)	9,900 (9,500–10,200)	3.2 (3.1–3.3)
**Uruguay**	28 (28–29)	269 (266–272)	88 (82–93)	25,700 (24,000–27,400)	4.0 (3.7–4.2)
**Venezuela**	241 (235–247)	237 (231–243)	139 (90–188)	4,200 (2,800–5,700)	3.8 (2.4–5.1)

^**i**^**.** The number of DALYs was estimated by assuming that the annual growth rate of DALYs equals the average growth rate observed during the period 2009–2019.

^**ii**^**.** The number of DALYs per 1,000 people was estimated by dividing the projected total number of DALYs by the total population in the period 2020–2050.

^**iii**^**.** Per capita GDP loss is relative to each country’s average population in the period 2020–2050. Values are approximated to the nearest hundred.

^**iv**^**.** Loss as a percentage of total GDP during the period 2020–2050 was estimated by dividing the total GDP loss due to NMHs by the discounted sum of annual GDP in the status quo scenario over the period 2020–2050.

The total economic burden of NMHs over the period 2020–2050 is substantial in all countries, ranging from $88 billion in Uruguay (95% CI: $82–$93 billion) to $3.7 trillion in Brazil (95% CI: $3.5–$3.9 trillion) (2022 international $). The highest output losses are in Brazil, Argentina, and Colombia. These are also the countries with the largest expected health burden of NMHs as measured in total DALYs and the countries with the largest populations in the region (Table A2 in S1 Appendix). Overall, the total economic burden of NMHs in the South American region amounts to $7.3 trillion (95% CI: $6.8–$7.8 trillion).

The total economic burden is considerably larger than the amount countries spend annually for healthcare (Table A2 in S1 Appendix). Based on the most recent estimates of healthcare spending (2019 data), South American countries devote about 5–10% of their annual GDP to healthcare. The total estimated GDP losses amount to 10 to 15 times current annual health spending. For example, in Peru annual health expenditures amount to 5.2% of GDP, while the expected economic burden of NMHs is 14 times greater, at 75% of 2021 GDP. At the other end of the ranking, health spending in Brazil is 9.6% of GDP, and the economic burden of NMHs is 10 times greater, at about 99% of 2021 GDP.

Once we adjust for population size, the largest economic burden of NMHs is in Chile, followed by Uruguay and Argentina. The average per capita output losses are, respectively, $27,300 in Chile (95% CI: $25,900–$28,800), $25,700 in Uruguay (95% CI: $24,000–$27,400), and $23,900 in Argentina (95% CI: $22,000–$25,700). These countries have the largest GDP per capita and among the largest healthcare spending per capita (Table A2 in S1 Appendix). As a result, they gain larger benefits from averting each death or nonfatal case of illness. Chile, Uruguay, and Argentina are also characterized by large per capita health burdens of NMHs. This partly explains the high economic burden in per capita terms.

The last column of Table 2 shows the economic burden of NMHs as a percentage of total GDP over the period 2020–2050. This measure accounts for baseline differences in GDP and in growth potential. The estimates suggest that the economic burden of NMHs equals an annual income tax of around 4%. Stated differently, if all NMHs were eliminated at no cost, annual GDP in South American countries over the period 2020–2050 would be about 4% larger.

The largest burdens as a percentage of total GDP over the period 2020–2050 are in Brazil (4.5%, 95% CI: 4.2%–4.7%), Chile (4.4%, 95% CI: 4.1%–4.6%), and Argentina (4.4%, 95% CI: 4.0%–4.7%), while the lowest economic burden is in Peru (3.2%, 95% CI: 3.1%–3.3%). Several factors drive differences in country-level results, including the underlying disease burden, per capita GDP, population size, and healthcare costs. The relatively low per capita health burden likely explains the low burden in Peru, while the magnitude of healthcare costs likely drives the large burdens in Chile and Brazil.

To put these numbers in perspective (Table A3 in S1 Appendix), note that the estimated annual increase in GDP following the hypothetical elimination of NMHs is close to what countries spend annually on education. For instance, in 2020 Paraguay spent 3.3% of GDP on education; the GDP gain from NMH elimination (4.3%) could cover and increase education expenses. The GDP gain is also about fourfold what Argentina and Brazil spent in 2021 on public and publicly guaranteed debt service (respectively, 1.2% and 1.4%) and is larger than Colombia’s annual military expenditures (3.4% in 2021). The annual increase in GDP following the elimination of NMHs is approximately a quarter of the 2020 tax revenues in Chile (16.2%), Colombia (14.2%), Ecuador (12.5%), Peru (13.2%) and Uruguay (18.6%). Thus, eliminating NMHs would represent a considerable source of potential revenues for these countries.

The projected economic burden in Venezuela is the most uncertain: The mean estimate is $139 billion (3.8% of GDP in 2020–2050), with a 95% confidence interval ranging from $90 billion to $188 billion (2.4%–5.1% of GDP in 2020–2050). The wide range of estimates is due to the high volatility of past GDP growth rates estimated from the data.

**Fig 2:**
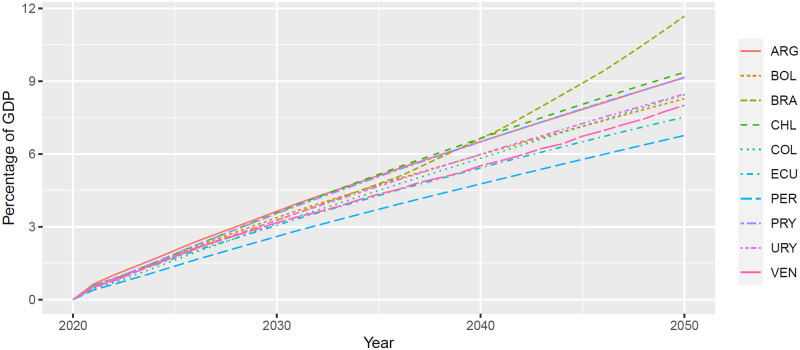
Macroeconomic burden of NMHs by country and year (as a percentage of yearly GDP).

Delaying measures to curb the prevalence of NMHs will have long-term compound negative effects. Fig 2 displays the yearly macroeconomic burden of NMHs. For each country and year, we compared the GDP in the status quo and the GDP in a counterfactual scenario with no NMHs and computed the increase in yearly GDP as a percentage of the status quo GDP if NMHs were eliminated. The figure shows that all countries would gain considerably from reducing the burden of NMHs. In addition, because prevalence of NMHs affects the economic growth process, long-term gains are larger than short-term gains.

Table 3 presents the economic burden attributable to the leading NMHs: cardiovascular diseases, diabetes and kidney diseases, chronic respiratory conditions, cancers, and mental health conditions. As previously, we assume a 3% annual discount rate and disease burden based on the mean estimates from GBD [1]. The economic burden is measured in terms of total GDP loss (2022 international $). Table A4 in S1 Appendix reports the economic burden in terms of percentage of total GDP over the period 2020–2050.

**Table 3:** Macroeconomic burden of leading NMHs: Total GDP loss (in billions, 2022 international $)^i^.

Country	Cancers	Cardiovascular diseases	Chronic respiratory diseases	Diabetes and kidney diseases	Mental health conditions
**Argentina**	$151 (142–161)	$104 (100–107)	$43 (41–45)	$87 (83–92)	$80 (74–85)
**Bolivia**	$10 (9–11)	$7 (7–8)	$3 (2–3)	$8 (8–9)	$8 (7–9)
**Brazil**	$275 (264–287)	$302 (293–311)	$96 (93–99)	$241 (231–252)	$272 (255–289)
**Chile**	$55 (52–48)	$39 (38–40)	$16 (15–17)	$48 (47–50)	$54 (52–56)
**Colombia**	$73 (70–76)	$55 (53–56)	$20 (20–21)	$86 (83–89)	$63 (61–66)
**Ecuador**	$20 (19–20)	$17 (16–17)	$4 (3–4)	$16 (16–16)	$10 (10–10)
**Paraguay**	$9 (8–9)	$9 (8–9)	$3 (3–3)	$10 (9–10)	$11 (11–11)
**Peru**	$33 (32–33)	$22 (21–22)	$8 (8–9)	$29 (28–30)	$26 (25–27)
**Uruguay**	$12 (11–13)	$7 (7–7)	$3 (3–3)	$6 (6–7)	$7 (7–8)
**Venezuela**	$14 (11–18)	$14 (12–16)	$3 (3–4)	$17 (14–20)	$10 (7–13)

^**i**^**.** 95% confidence intervals are in parentheses. Values are approximated to the nearest billion.

Among the five conditions considered in Table 3, cancers are the leading contributor of macroeconomic burden in most countries. The expected cost of cancers ranges from $275 billion in Brazil (95% CI: $264–$287) to $9 billion in Paraguay (95% CI: $8–$9) (2022 international $). As a percentage of GDP in the period 2020–2050 (Table A4 in S1 Appendix), the economic burden of cancers ranges from 0.28% in Peru (95% CI: 0.27%–0.28%) to 0.57% in Argentina (95% CI: 0.53%–0.60%). In Brazil, the largest economic burden is due to cardiovascular diseases, while in Colombia and Venezuela the leading causes of macroeconomic loss are diabetes and kidney diseases. Mental health conditions contribute substantially to the macroeconomic cost of NMHs, especially in Paraguay, with an economic burden equal to 0.44% of 2020–2050 GDP (95% CI: 0.43%–0.45%). The high burden associated with cancers is in part due to its age structure and the fact that, compared with other conditions, cancer causes a relatively high health burden among working-age individuals. Our results about the burden of cancers are also in line with a recent similar macroeconomic study that estimates that 29 cancers will cost Latin America and the Caribbean region 0.33% of annual GDP between 2020 and 2050 (with a 3% discount rate) [18].

**Fig 3:**
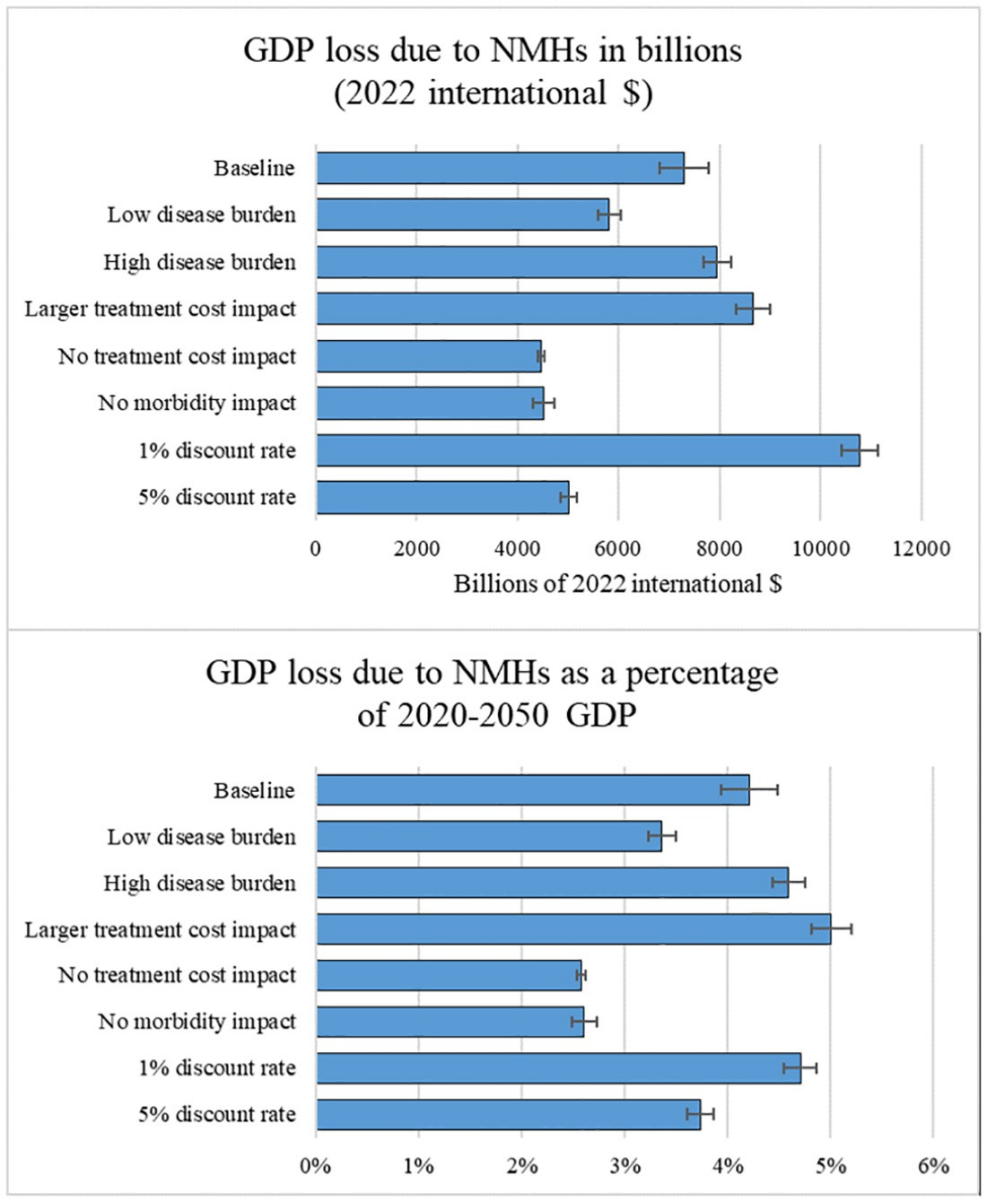
Macroeconomic burden of NMHs for the whole South American region over the period 2020–2050 across various scenarios. *Baseline scenario* = 3% discount rate, disease burden based on median estimates from the 2019 Global Burden of Diseases (GBD) [1], morbidity affects labor force participation, and treatment costs decrease investments in physical capital proportionally to the saving rate. *Low disease burden* = disease burden based on 2.5 percentile estimates provided by GBD; all other assumptions as in the baseline scenario. *High disease burden* = burden based on 97.5 percentile estimates provided by GBD; all other assumptions as in the baseline scenario. *Larger treatment cost impact scenario* = treatment costs reduce physical capital investments by a proportion 50% greater than the saving rate; all other assumptions as in the baseline scenario. *No treatment cost impact scenario* = treatment costs do not reduce physical capital investments; all other assumptions as in the baseline scenario. *No morbidity impact* = morbidity does not affect labor force participation; all other assumptions as in the baseline scenario. *1% discount rate scenario* = 1% discount rate; all other assumptions as in the baseline scenario. *5% discount rate scenario* = 5% discount rate; all other assumptions as in the baseline scenario. The error bars denote 95% confidence intervals.

Fig 3 investigates the sensitivity of our results to some of the model assumptions. The figure shows the macroeconomic burden of NMHs for the South American region under different scenarios. Two different measures of macroeconomic burden are depicted: expected GDP loss due to NMHs in billions of 2022 international dollars and expected GDP loss due to NMHs as a percentage of GDP over the period 2020–2050. The regional GDP loss was computed by summing country-specific estimates of GDP loss. We then divided that sum by the sum of country-specific estimates of GDP over the period 2020–2050 to determine the percentage estimates. The *baseline scenario* corresponds to the results depicted in Table 2, with a 3% discount rate and a disease burden based on median estimates from GBD [1]. It also assumes that NMH-related morbidity negatively affects labor force participation and that NMH-related treatment costs decrease investments in physical capital by a proportion equal to the saving rate. All other scenarios modify a single assumption used in the baseline scenario. In the *low disease burden scenario*, we adopt disease burden estimates based on the 2.5 percentile estimates provided by GBD [1]. In the *high disease burden scenario*, we adopt disease burden estimates based on the 97.5 percentile estimates provided by GBD [1]. In the *larger treatment cost impact scenario*, we assume that the proportion of treatment costs financed through savings is larger than the saving rate (a 50% increase). In the *no treatment cost impact scenario*, we assume that treatment costs do not affect physical capital accumulation, while in the *no morbidity impact scenario* we assume that morbidity does not affect labor supply. Finally, in the *1% discount rate scenario* and in the *5% discount rate scenario* we modify the discount rate. Tables A5 and A6 in S1 Appendix present country-specific results for all these scenarios.

In the baseline scenario, the regional macroeconomic loss due to NMHs is $7.3 trillion (2022 international $) (95% CI: $6.8–$7.8 trillion), or 4.2% of regional GDP over 2020–2050 (95% CI: 3.9%–4.5%). Compared with the baseline scenario, when using the high disease burden estimates, the economic costs of NMHs for the South American region amounts to about $8 trillion (2022 international $) (95% CI: $7.7–$8.2 trillion), or 4.6% (95% CI: 4.4%–4.8%) of regional GDP in the period 2020–2050. In contrast, if we use the low disease burden estimates, the economic costs of NMHs are equivalent to an annual income tax of 3.4% (95% CI: 3.3%–3.5%). Increasing the proportion of treatment costs financed through savings substantially increases the macroeconomic burden of NMHs. In this scenario, the macroeconomic loss due to NMHs amounts to $8.7 trillion (95% CI: $8.3–$9.0), which is equivalent to 5% (95% CI: 4.8%–5.2%) of regional GDP in the period 2020–2050. Note that the larger the proportion of treatment costs financed through savings, the more resources used in treatments could otherwise have been invested in physical capital. In a scenario where treatment costs do not affect physical capital accumulation, the macroeconomic burden of NMHs amounts to $4.5 trillion or 2.6% of regional 2020–2050 GDP (with small confidence intervals). The large decline compared to the baseline scenario highlights the importance of the treatment costs channel (i.e., the negative impact of treatment costs on physical capital accumulation) on the overall burden of NMHs. Note that the relative importance of the treatment costs channel varies across countries (Table A5 in S1 Appendix). For instance, in Chile and Ecuador, the treatment cost channel explains less than 15% of the overall macroeconomic burden of NMHs. In contrast, in Colombia, Paraguay and Uruguay the treatment cost channel explains about 40% of the macroeconomic burden. If we neglect to consider the impact of morbidity on labor supply, the economic burden of NMHs remains substantial, amounting to $4.5 trillion (95% CI: $4.3–$4.7 trillion) or 2.6% (95% CI: 2.5%–2.7%) of regional 2020–2050 GDP. Finally, we vary the discount rate. Even with a higher discount rate (5% instead of 3%), the economic burden of NMHs is around $5 trillion (95% CI: $4.8–$5.2 trillion), and it corresponds to an annual income tax around 3.7% (95% CI: 3.6%–3.8%). A lower discount rate (1% instead of 3%) is posited to increase the macroeconomic burden of NMHs, with a regional loss equal to $10.8 trillion (95% CI: $10.4–$11.1 trillion) and an annual income tax of 4.7% (95% CI: 4.5%–4.9%). Note that, with a lower discount rate, the regional GDP over the period 2020–2050 increases as well, as it corresponds to the discounted sum of annual GDP in the status quo scenario. As a consequence, the loss in monetary terms is considerably larger than the loss in percentage terms compared with the baseline scenario.

Finally, we compare our estimates of economic burden of NMHs with the results derived from alternative economic evaluation approaches, i.e., the COI method and the VSL method. In the COI approach, the burden of a disease is given by the sum of its direct and indirect costs [30], while the VSL approach is based on estimates on individuals’ willingness to trade small changes in income for changes in fatal and non-fatal risks [45]. Compared with the COI and VSL approaches, the macroeconomic approach leads to conservative estimates of the burden of disease (Table A7 in S1 Appendix). In particular, the economic burdens with COI and VSL are, respectively, about two and five times larger than the burden computed with the macroeconomic model. The VSL approach captures both the intrinsic and instrumental value of being alive and in good health, while the macroeconomic model captures only (part of) the instrumental value. This explains why the VSL estimates are considerably larger than the macroeconomic ones. The assumed large treatment costs of NHMs in large part drive the COI estimates. In the macroeconomic model, treatment costs are detrimental insofar as they prevent investment in physical capital. Since, by assumption, only a fraction of treatment costs would otherwise be invested in physical capital, the macroeconomic loss due to NMHs is less sensitive to treatment costs than the COI estimates. In addition, the COI approach fails to properly account for differences in human capital across the population and for the impact of health on effective labor supply.

## Discussion

In the paper, we adopt a macroeconomic model to project the GDP loss caused by NMHs in South America over the period 2020–2050. Our estimates of macroeconomic burden account for the effect of NMH-related mortality and morbidity on age-specific labor force participation rates and for the impact of NMH-related health expenditures on physical capital accumulation. We also control for age-differences in human capital. For a given level of educational attainment, human capital increases with age because workers’ experience increases with age. We show that, even though the risk of NMHs concentrates in older ages, the negative impact of NMHs on labor supply is substantial, due both to appreciable numbers of working-age individuals affected by NMHs and to their level of human capital.

The burden of NMHs is particularly high in South America and is likely to increase over time due to population aging and the high prevalence of many controllable risk factors for NMHs (e.g., smoking, alcohol consumption, and physical inactivity). Overall, the population share aged 65+ in the region will double over the next three decades (from 9% to 20%), and the median age will increase from 30 years to 40 years (Table A8 in S1 Appendix). Because age is the main predictor of chronic conditions, the share of the population with NMHs in South America is expected to increase appreciably over time. South American countries also record relatively high (often higher than global averages) rates of tobacco use, harmful use of alcohol, physical inactivity, and diets that are high in calories, sugar, and saturated fats (Table A9 in S1 Appendix). In particular, the percentage of overweight adults in the region is around 60%, compared with a global average of 39%, and is rapidly increasing over time. This contributes to the projected high economic burden of NMHs in the region.

We also show that countries vary little in terms of macroeconomic burden (from 3.2% in Peru to 4.5% in Brazil). Although the 10 countries share relatively high burdens of NMHs, a fair amount of heterogeneity exists in terms of demographics, population health, and economic development (Table A2 in S1 Appendix). Based on the World Bank income classification, the 10 countries include lower-middle income (Bolivia), upper-middle income, and high-income countries (Chile and Uruguay), with Venezuela currently not classified by World Bank income group. Life expectancy at birth ranges from 64 in Bolivia to 79 in Chile, while life expectancy at age 65 ranges from 11 years to 19 years in those same countries. Even though all countries are experiencing rapid population aging, the projected share of individuals age 65+ in 2050 ranges from 9.3% in Bolivia to 25.8% in Chile. This heterogeneity explains the difference in the estimated economic burden of NMHs across countries.

In the paper, we have estimated the benefits of complete elimination of NMHs. While our figures cannot be directly used to identify the most cost-beneficial prevention or treatment interventions, our results raise awareness about the broad social and economic benefits of reducing the burden of NMHs. In addition, our results can be used to promote interventions that reduce the risk factors for NMHs. Future work can be tailored to specific health interventions and to estimating the macroeconomic benefits of partial elimination of NMHs.

Our analysis points to the broad benefits of health interventions beyond reductions in mortality, morbidity, and healthcare costs. These benefits take the form of increased labor force participation and productivity, enhanced learning abilities and higher educational attainment, and possibly more equity and social cohesion (under the premise that disease burden is larger among disadvantaged groups). The COVID-19 pandemic illustrates in stark terms the myriad economic and social implications of diseases [46]. Nevertheless, standard health technology assessment methods tend to neglect these broad benefits in economic valuation, focusing instead on a relatively narrow set of health-centric benefits [47, 48]. The chosen evaluation method has important implications for resource allocations and funding of health interventions. If health interventions are routinely undervalued, then the resources devoted to promoting health will be less than optimal. Thus, our study advocates accounting for the full societal benefits of health improvements.

While our paper focuses on the macroeconomic costs of NMHs, other methods can capture (at least part of) the broad costs of diseases. We compare our macroeconomic estimates with the results of a COI study and a VSL analysis, and we find that our approach leads to conservative estimates of the economic burden of NMHs in South America. We argue that the COI method may lead to biased estimates of the economic burden of diseases because it fails to account for demographic changes and the impact of health on physical and human capital accumulation. In principle, the VSL approach incorporates both the intrinsic and the instrumental values of good health, thereby offering a more complete picture of the overall burden of a disease. However, its practical implementation faces several challenges related to the lack of studies in low- and middle-income countries, debates about the best methodology to elicit willingness to pay, and the sensitivity of willingness-to-pay measures to the type of risk and to the income and age of respondents [45]. In the absence of VSL studies focusing on South American countries, we adopted the conventional assumption that VSL is proportional to a country’s per capita GDP. However, the validity of this assumption is uncertain. In addition, this assumption raises ethical concerns because it implies that saving lives in wealthy countries is more valuable than saving lives in less-wealthy countries. The adoption of social welfare function methods and equity weights can overcome these shortcomings [49].

We believe that the macroeconomic burden of NMHs is a useful and easily interpretable metric of the economic costs of NMHs. Moreover, it is a metric that speaks directly to ministers of finance, who decide the allocation of resources across different ministries. Thus, estimates of the macroeconomic benefits of NMH interventions could be easily compared with, say, the macroeconomic effects of transportation or education policies.

The analysis has several limitations. We lacked data on the impact of specific diseases on labor force participation for the countries under study. Therefore, we proxy the effect of morbidity on labor supply with the contribution of morbidity to the health burden of NMHs (measured in DALYs). In addition, we assume that morbidity affects the probability of participating in the labor force but not individuals’ productivity and human capital. For instance, long periods out of the labor force due to ill health may cause human capital to depreciate. In addition, because of data constraints, we assume independence of mortality and morbidity across diseases, and we do not account for the broad health impacts of eliminating a specific disease (e.g., eliminating one disease may reduce the likelihood of other diseases). When we simulate the effects of complete elimination of all NMHs, the independence assumption is less of a concern because we rely on aggregate data of NMH prevalence, mortality and morbidity. The independence assumption is more problematic when we simulate the macroeconomic burden of eliminating a single disease because we do not estimate the spillover effects on the mortality and morbidity of other diseases. Note that the sign of this spillover effect is a priori ambiguous. On the one hand, reducing the prevalence of one disease (e.g., diabetes) is expected to reduce the prevalence of other correlated diseases (e.g., cardiovascular diseases). On the other hand, individuals may engage in more risky behaviors as a response to reductions in disease burden (e.g., if there is a drug that prevents cancer, individuals may adopt a less healthy lifestyle). Estimates of these spillover effects are a fruitful venue for future research.

Reducing the burden of NMHs would also have sizable implications in terms of formal and informal care, but we neglect to include this in the analysis. For example, family caregivers may withdraw from the labor market [50, 51]. Also, the cost data we use in the analysis capture the costs of treating NMHs, but they do not account for the cost of formal and informal care. Moreover, NMHs impose substantial financial costs on households in terms of catastrophic out-of-pocket health expenditures and household income losses [52], especially in economies with gender-specific division of labor (e.g., if only the household head works, the death or illness of the household head has catastrophic consequences for household finances). Reliance on family members to provide care can also increase the mental health burden of NMHs [53]. The analysis neglects to consider the interplay between NMHs and household decision making (e.g., regarding the labor supply of household members or the provision of informal care), and the resulting effect on economic growth.

Additionally, we do not incorporate general equilibrium effects in the model. For example, we do not account for individual compensatory behaviors, i.e., how individuals respond to the elimination of diseases by, e.g., saving more or investing more in education. While adopting a general equilibrium model would improve the estimates, model calibration would require more restrictive assumptions that are not always fully verifiable through data.

Finally, we measure the economic burden of NMHs in terms of GDP impacts. However, GDP is broadly agreed to be an imperfect measure of the wellbeing of a country and its people [54]. For instance, GDP does not account for productive nonmarket contributions, e.g., childcare and volunteering. In particular, this leads to the undervaluation of health among older people [55]. Furthermore, GDP measures do not account for various dimensions of inequality (e.g., income, race/ethnicity, and gender) or for the distributional implications of health interventions. Future work can address these shortcomings.

## Conclusion

The paper estimates the economic burden of NMHs over the period 2020–2050 in South America. We measure economic burden in terms of GDP losses, and we adopt a previously validated macroeconomic model to project such a burden [12]. We find that the macroeconomic burden of NMHs is substantial. Our central estimates suggest that the overall burden of NMHs in the region amounts to $7.3 trillion (2022 international $) over 2020–2050 (with a 3% discount rate). Overall, the macroeconomic burden is around 4% of total GDP over 2020–2050. In other words, without NMHs, annual GDP over 2020–2050 would be about 4% larger in all countries. This number corresponds roughly to what countries are now spending annually on education. The results of the paper strongly justify investment in NMH prevention, early detection, treatment, and formal and informal care.

## Supporting information

S1 Appendix The Macroeconomic burden of noncommunicable diseases and mental health conditions in South America: Supplementary materials. (DOCX)
