# Supplementary material for: The macroeconomic burden of noncommunicable diseases and mental health conditions in South America
Source: PLoS One. 2023 Oct 20;18(10):e0293144. doi: 10.1371/journal.pone.0293144 (PMC10588886; doi:10.1371/journal.pone.0293144)
Supplement: S1 Appendix — (DOCX) [file pone.0293144.s001.docx]

**The Macroeconomic Burden of Noncommunicable Diseases**

**and Mental Health Conditions in South America:**

**Supplementary Materials**

Maddalena Ferranna, Daniel Cadarette, Simiao Chen,

Parastou Ghazi, Faith Ross, Leo Zucker, David E. Bloom

**Contents:**

1. Additional tables …………..……………………………………………….....2
2. Description of the macroeconomic model………………………………….... 8
3. Calibration of the macroeconomic model………………………………….... 10
4. Conversion to 2022 international $.................................................................. 11
5. Alternative evaluation methods………...……………………………………. 11

**A. Additional Tables**

**Table A1. Prevalence rates, mortality rates, deaths, and disability-adjusted life years (DALYs) due to noncommunicable diseases and mental health conditions (NMHs) in South America.**

| **Disease** | **Prevalence rate (per 100,000)** | **Mortality rate (per 100,000)** | **Total deaths (thousands)** | **Total DALYs (millions)** |
| --- | --- | --- | --- | --- |
| Cancers | 921 | 127 | 547 | 13.6 |
| Cardiovascular diseases | 5,391 | 175 | 754 | 15.8 |
| Chronic respiratory diseases | 6,930 | 34 | 145 | 3.8 |
| Diabetes and kidney diseases | 12,508 | 51 | 221 | 7.5 |
| Mental health conditions | 54,881 | 36 | 153 | 16.9 |
| All NMHs | 91,862 | 483 | 2,081 | 86.7 |

***Notes:*** Data are from the Global Burden of Diseases 2019 [1]. Due to data availability, the figures refer to the following group of countries: Argentina, Bolivia, Brazil, Chile, Colombia, Ecuador, Guyana, Peru, Paraguay, Suriname, Uruguay, and Venezuela.

**Table A2. Economic, demographic, and health indicators.**

| **Country** |  | **GDP per capita, PPP (constant 2017 international $)^ii^** | **Population in 2020 (millions)^iii^** | **Life expectancy at birth^iii^** | | **Life expectancy at age 65^iii^** | | **Health expenditures as a percentage of GDP (%)^iv^** |
| --- | --- | --- | --- | --- | --- | --- | --- | --- |
|  | **Total GDP, PPP (billions, constant 2017 international $)^i^** |  |  |  |  |  |  |  |
|  |  |  |  | **2020** | **2050** | **2020** | **2050** |  |
| **Argentina** | 986 | 21,527 | 45.0 | 75.9 | 82.4 | 17.1 | 21.0 | 9.5 |
| **Bolivia** | 97 | 8,052 | 11.9 | 64.5 | 73.6 | 11.1 | 15.0 | 6.9 |
| **Brazil** | 3,128 | 14,592 | 213.2 | 74.0 | 81.3 | 16.4 | 20.0 | 9.6 |
| **Chile** | 496 | 25,449 | 19.4 | 79.4 | 85.7 | 19.1 | 23.3 | 9.3 |
| **Colombia** | 755 | 14,649 | 50.9 | 74.8 | 82.3 | 16.7 | 20.9 | 7.7 |
| **Ecuador** | 190 | 10,669 | 17.6 | 72.2 | 82.7 | 15.1 | 21.5 | 7.8 |
| **Paraguay** | 92 | 13,688 | 6.6 | 73.2 | 77.6 | 16.5 | 18.4 | 7.2 |
| **Peru** | 422 | 12,515 | 33.3 | 73.7 | 81.6 | 15.9 | 20.3 | 5.2 |
| **Uruguay** | 78 | 22,801 | 3.4 | 78.4 | 82.4 | 19.1 | 21.2 | 9.4 |
| **Venezuela** | 269^v^ | 7,704^v^ | 28.5 | 71.1 | 77.6 | 15.7 | 18.2 | 5.4 |

***Notes:*** PPP=purchasing power parity. **i.** Gross domestic product (GDP) in 2021, World Bank [2]. Values are approximated to the nearest billion. **ii.** GDP per capita in 2021, World Bank [3]. **iii.** 2022 World Population Prospects [4]. **iv.** 2019 estimates, World Bank [5]. **v.** 2018 estimate from the Central Intelligence Agency’s Factbook [6].

**Table A3. Comparing the macroeconomic burden of NMHs to country-specific sources of revenues and expenditures.**

| **Country** | **Annual economic burden of NMHs (% of GDP in 2020-2050)^i^** | **Government expenditures on education in 2020 (% GDP)^ii^** | **Tax revenues in 2020 (% of GDP)^iii^** | **Military expenditures in 2021 (% of GDP)^iv^** | **Public and publicly guaranteed debt service in 2021 (% of GDP)^v^** |
| --- | --- | --- | --- | --- | --- |
| **Argentina** | 4.4 | 5.0 | 10.9 | 0.6 | 1.2 |
| **Bolivia** | 4.0 | 9.8 | 39.9^vi^ | 1.5 | 2.6 |
| **Brazil** | 4.5 | 6.0^vii^ | 13.0 | 1.2 | 1.4 |
| **Chile** | 4.4 | 5.6^vii^ | 16.2 | 2.0 | (missing) |
| **Colombia** | 3.9 | 4.9 | 14.2 | 3.4 | 4.2 |
| **Ecuador** | 3.7 | 4.1 | 12.5 | 2.5 | 2.9 |
| **Paraguay** | 4.3 | 3.3 | 9.5 | 1.0 | 2.3 |
| **Peru** | 3.2 | 4.2 | 13.2 | 1.1 | 0.7 |
| **Uruguay** | 4.0 | 4.6 | 18.6 | 2.3 | (missing) |
| **Venezuela** | 3.8 | 1.3^vi^ | 44.2^vi^ | 5.2^vii^ | (missing) |

***Notes:* i.** Estimates of the macroeconomic burden of NMHs as a percentage of GDP over the period 2020–2050 are derived from Table 2 in the main text. These are mean estimates. **ii.** World Bank data [7]. **iii.** World Bank data [8]. **iv.** World Bank data [9]. **v.** The World Bank provides data on public and publicly guaranteed debt service as a percentage of gross national income (GNI) [10]. To be consistent with the other measures, we converted those metrics of public and publicly guaranteed debt service as a percentage of GDP using country-specific ratios of 2021 GDP [2] versus 2021 GNI [11]. **vi.** 2017 estimate from the Central Intelligence Agency’s Factbook [6]. **vii.** 2019 estimate from the Central Intelligence Agency’s Factbook [6].

**Table A4. Macroeconomic burden of leading NMHs: Loss as a percentage of total GDP during 2020–2050.**

| **Country** | **Cancers** | **Cardiovascular diseases** | **Chronic respiratory diseases** | **Diabetes and kidney diseases** | **Mental health conditions** |
| --- | --- | --- | --- | --- | --- |
| **Argentina** | 0.57%  (0.53–0.60) | 0.39%  (0.38–0.40) | 0.16%  (0.15–0.17) | 0.33%  (0.31–0.34) | 0.30%  (0.28–0.32) |
| **Bolivia** | 0.36%  (0.33–0.40) | 0.27%  (0.26–0.29) | 0.09%  (0.09–0.10) | 0.30%  (0.28–0.32) | 0.30%  (0.27–0.33) |
| **Brazil** | 0.33%  (0.32–0.34) | 0.36%  (0.35–0.37) | 0.12%  (0.11–0.12) | 0.29%  (0.28–0.30) | 0.33%  (0.31–0.35) |
| **Chile** | 0.42%  (0.40–0.44) | 0.30%  (0.30–0.31) | 0.12%  (0.12–0.13) | 0.37%  (0.36–0.39) | 0.42%  (0.40–0.43) |
| **Colombia** | 0.33%  (0.32–0.35) | 0.25%  (0.25–0.26) | 0.09%  (0.09–0.10) | 0.39%  (0.38–0.41) | 0.29%  (0.28–0.30) |
| **Ecuador** | 0.37%  (0.36–0.38) | 0.32%  (0.31–0.32) | 0.07%  (0.07–0.07) | 0.30%  (0.30–0.31) | 0.19%  (0.18–0.19) |
| **Paraguay** | 0.33%  (0.32–0.34) | 0.34%  (0.33–0.35) | 0.11%  (0.11–0.11) | 0.38%  (0.37–0.38) | 0.44%  (0.43–0.45) |
| **Peru** | 0.28%  (0.27–0.28) | 0.18%  (0.18–0.19) | 0.07%  (0.07–0.07) | 0.25%  (0.24–0.25) | 0.22%  (0.22–0.23) |
| **Uruguay** | 0.54%  (0.51–0.57) | 0.32%  (0.31–0.33) | 0.15%  (0.15–0.16) | 0.29%  (0.28–0.31) | 0.33%  (0.32–0.35) |
| **Venezuela** | 0.38%  (0.29–0.49) | 0.38%  (0.33–0.43) | 0.09%  (0.07–0.10) | 0.45%  (0.37–0.54) | 0.27%  (0.20–0.35) |

***Notes:*** 95% confidence intervals are in parentheses.

**Table A5. Macroeconomic burden of NMHs as GDP loss in billions of 2022 international $ across different scenarios.**

| **Country** | **Baseline** | **Low disease burden** | **High disease burden** | **Larger treatment cost impact** | **No treatment cost impact** | **No morbidity impact** | **1% discount rate** | **5% discount rate** |
| --- | --- | --- | --- | --- | --- | --- | --- | --- |
| **Argentina** | 1,162  (1,071–1,253) | 1,013  (969–1,057) | 1,302  (1,254–1,351) | 1,395  (1,332–1,458) | 684  (673–696) | 729  (690–769) | 1,700  (1,633–1,768) | 808  (776–840) |
| **Bolivia** | 109  (95–123) | 88  (82–95) | 129  (121–137) | 133  (123–143) | 59  (57–60) | 72  (66–79) | 159  (149–170) | 76  (70–81) |
| **Brazil** | 3,702  (3,472–3,932) | 2,788  (2,680–2,896) | 3,720  (3,599–3,841) | 4,292  (4,132–4,452) | 2,495  (2,464–2,526) | 2,248  (2,149–2,348) | 5,515  (5,343–5,688) | 2,528  (2,447–2,609) |
| **Chile** | 566  (536–595) | 506  (492–521) | 633  (617–649) | 714  (693–735) | 263  (259–267) | 374  (361–387) | 832  (810–854) | 391  (381–402) |
| **Colombia** | 849  (804–894) | 694  (672–716) | 1,039  (1,009–1,069) | 1,049  (1,017–1,081) | 439  (433–445) | 535  (515–554) | 1,251  (1,217–1,284) | 585  (569–602) |
| **Ecuador** | 192  (187–197) | 149  (147–151) | 247  (242–253) | 218  (215–220) | 140  (138–142) | 101  (100–102) | 281  (277–285) | 134  (132–135) |
| **Paraguay** | 110  (107–114) | 91  (90–93) | 136  (134–139) | 137  (135–139) | 56  (55–57) | 75  (73–76) | 162  (160–165) | 76  (75–78) |
| **Peru** | 376  (362–389) | 306  (300–311) | 454  (445–463) | 459  (450–468) | 207  (203–210) | 228  (223–232) | 552  (542–562) | 260  (255–264) |
| **Uruguay** | 88  (82–93) | 78  (75–81) | 99  (95–102) | 108  (104–112) | 45  (44–46) | 57  (54–59) | 129  (124–133) | 61  (59–63) |
| **Venezuela** | 139  (90–188) | 107  (85–129) | 197  (167–227) | 168  (134–202) | 81  (74–88) | 94  (73–115) | 204  (167–241) | 97  (80–114) |

***Notes***: *Baseline scenario* = 3% discount rate, disease burden based on median estimates from the Global Burden of Diseases 2019 (GBD) [1], morbidity affects labor force participation, and treatment costs decrease investments in physical capital proportionally to the saving rate. *Low disease burden* = disease burden based on 2.5 percentile estimates provided by GBD; all other assumptions as in the baseline scenario. *High disease burden* = burden based on 97.5 percentile estimates provided by GBD; all other assumptions as in the baseline scenario. *Larger treatment cost impact scenario* = treatment costs reduce physical capital investments by a proportion 50% greater than the saving rate; all other assumptions as in the baseline scenario. *No treatment cost impact scenario* = treatment costs do not reduce physical capital investments; all other assumptions as in the baseline scenario. *No morbidity impact* = morbidity does not affect labor force participation; all other assumptions as in the baseline scenario. *1% discount rate scenario* = 1% discount rate; all other assumptions as in the baseline scenario. *5% discount rate scenario* = 5% discount rate; all other assumptions as in the baseline scenario. 95% confidence intervals are in parentheses.

**Table A6. Macroeconomic burden of NMHs as a percentage of GDP over the period 2020–2050 across different scenarios.**

| **Country** | **Baseline** | **Low disease burden** | **High disease burden** | **Larger treatment cost impact** | **No treatment cost impact** | **No morbidity impact** | **1% discount rate** | **5% discount rate** |
| --- | --- | --- | --- | --- | --- | --- | --- | --- |
| **Argentina** | 4.4  (4.0–4.7) | 4.5  (4.3–4.7) | 5.8  (5.6–6.0) | 6.2  (5.9–6.5) | 3.1  (3.0–3.1) | 3.3  (3.1–3.4) | 5.7  (5.5–6.0) | 4.7  (4.5–4.8) |
| **Bolivia** | 4.0  (3.5–4.5) | 3.9  (3.6–4.2) | 5.6  (5.3–6.0) | 5.8  (5.4–6.3) | 2.6  (2.5–2.6) | 3.2  (2.9–3.4) | 5.3  (4.9–5.6) | 4.3  (4.0–4.6) |
| **Brazil** | 4.5  (4.2–4.7) | 4.0  (3.8–4.1) | 5.3  (5.2–5.5) | 6.1  (5.9–6.4) | 3.6  (3.5–3.6) | 3.2  (3.1–3.4) | 6.0  (5.8–6.2) | 4.7  (4.5–4.8) |
| **Chile** | 4.4  (4.1–4.6) | 4.6  (4.5–4.8) | 5.8  (5.7–6.0) | 6.6  (6.4–6.7) | 2.4  (2.4–2.4) | 3.4  (3.3–3.6) | 5.8  (5.6–5.9) | 4.6  (4.5–4.7) |
| **Colombia** | 3.9  (3.7–4.1) | 3.8  (3.7–3.9) | 5.7  (5.5–5.8) | 5.7  (5.5–5.9) | 2.4  (2.4–2.4_ | 2.9  (2.8–3.0) | 5.1  (5.0–5.3) | 4.1  (4.0–4.2) |
| **Ecuador** | 3.7  (3.6–3.7) | 3.4  (3.3–3.4) | 5.6  (5.5–5.7) | 4.9  (4.9–5.0) | 3.2  (3.1–3.2) | 2.3  (2.2–2.3) | 4.8  (4.7–4.9) | 3.9  (3.8–4.0) |
| **Paraguay** | 4.3  (4.2–4.5) | 4.3  (4.2–4.3) | 6.3  (6.2–6.5) | 6.4  (6.3–6.5) | 2.6  (2.6–2.7) | 3.5  (3.4–3.5) | 5.7  (5.6–5.8) | 4.6  (4.5–4.7) |
| **Peru** | 3.2  (3.1–3.3) | 3.1  (3.0–3.1) | 4.6  (4.5–4.7) | 4.6  (4.6–4.7) | 2.1  (2.1–2.1) | 2.3  (2.2–2.3) | 4.2  (4.1–4.3) | 3.4  (3.3–3.5) |
| **Uruguay** | 4.0  (3.7–4.2) | 4.2  (4.1–4.4) | 5.3  (5.2–5.5) | 5.9  (5.6–6.1) | 2.4  (2.4–2.5) | 3.1  (2.9–3.2) | 5.3  (5.1–5.4) | 4.2  (4.1–4.4) |
| **Venezuela** | 3.8  (2.4–5.1) | 3.5  (2.7–4.2) | 6.4  (5.4–7.3) | 5.4  (4.3–6.5) | 2.6  (2.4–2.8) | 3.0  (2.3–3.7) | 5.0  (4.1–5.9) | 4.0  (3.3–4.7) |

***Notes***: *Baseline scenario* = 3% discount rate, disease burden based on median estimates from the 2019 Global Burden of Diseases Study (GBD) [1], morbidity affects labor force participation, and treatment costs decrease investments in physical capital proportionally to the saving rate. *Low disease burden* = disease burden based on 2.5 percentile estimates provided by GBD; all other assumptions as in the baseline scenario. *High disease burden* = burden based on 97.5 percentile estimates provided by GBD; all other assumptions as in the baseline scenario. *Larger treatment cost impact scenario* = treatment costs reduce physical capital investments by a proportion 50% greater than the saving rate; all other assumptions as in the baseline scenario. *No treatment cost impact scenario* = treatment costs do not reduce physical capital investments; all other assumptions as in the baseline scenario. *No morbidity impact* = morbidity does not affect labor force participation; all other assumptions as in the baseline scenario. *1% discount rate scenario* = 1% discount rate; all other assumptions as in the baseline scenario. *5% discount rate scenario* = 5% discount rate; all other assumptions as in the baseline scenario. 95% confidence intervals are in parentheses.

**Table A7. Economic burden of NMHs over the period 2020–2050 with alternative evaluation methods (billions, 2022 international $).**

| **Country** | **Macroeconomic model** | **Cost of illness** | **Value per statistical life** |
| --- | --- | --- | --- |
| **Argentina** | 1,162  (1,071–1,253) | 2,593  (2,561–2,625) | 6,357  (6,270–6,444) |
| **Bolivia** | 109  (95–123) | 223  (219–227) | 509  (506–512) |
| **Brazil** | 3,702  (3,472–3,932) | 8,705  (8,630–8,779) | 19,072  (18,944–19,201) |
| **Chile** | 566  (536–595) | 1,223  (1,207–1,238) | 2,943  (2,909–2,977) |
| **Colombia** | 849  (804–894) | 1,753  (1,736–1,770) | 4,133  (4,076–4,190) |
| **Ecuador** | 192  (187–197) | 449  (442–455) | 1,014  (999–1,029) |
| **Paraguay** | 110  (107–114) | 212  (209–215) | 507  (501–513) |
| **Peru** | 376  (362–389) | 693  (682–703) | 1,735  (1,715–1,755) |
| **Uruguay** | 88  (82–93) | 210  (208–212) | 586  (579–593) |
| **Venezuela** | 139  (90–188) | 227  (220–235) | 844  (818–871) |

***Notes:*** 3% discount rate. The value-per-statistical-life approach assumes that the value per statistical life year is equal to one-time GDP per capita.

**Table A8. Aging indicators.**

| **Country** | **Share 65+ (%)** | | **Share 85+ (%)** | | **Median age (years)** | |
| --- | --- | --- | --- | --- | --- | --- |
|  | **2020** | **2050** | **2020** | **2050** | **2020** | **2050** |
| **Argentina** | 11.7 | 19.1 | 1.4 | 2.8 | 31.0 | 39.9 |
| **Bolivia** | 5.0 | 9.3 | 0.2 | 0.5 | 23.7 | 31.7 |
| **Brazil** | 9.3 | 21.9 | 0.6 | 2.54 | 32.4 | 43.6 |
| **Chile** | 12.4 | 25.8 | 1.5 | 4.92 | 34.5 | 46.8 |
| **Colombia** | 8.5 | 20.9 | 0.5 | 2.73 | 30.4 | 43.2 |
| **Ecuador** | 7.5 | 17.0 | 0.6 | 2.19 | 27.2 | 38.6 |
| **Paraguay** | 6.2 | 12.5 | 0.5 | 1.32 | 25.3 | 33.5 |
| **Peru** | 8.3 | 16.6 | 0.6 | 1.73 | 28.0 | 37.3 |
| **Uruguay** | 15.3 | 23.2 | 2.5 | 4.22 | 35.0 | 44.5 |
| **Venezuela** | 8.0 | 14.7 | 0.4 | 1.42 | 27.8 | 36.0 |
| **South America** | **9.2** | **19.8** | **0.7** | **2.43** | **30.9** | **41.2** |

***Source:*** 2022 World Population Prospects [4].

**Table A9. Controllable risk factors.**

| **Country** | **Prevalence of current tobacco use 15+**  **(age-standardized %)^i^** | **Alcohol consumption per capita 15+ (liters of pure alcohol)^ii^** | **Prevalence of insufficient physical activity 18+**  **(age-standardized %)^iii^** | **Prevalence of overweight among adults 18+**  **(age-standardized %)^iv^** |
| --- | --- | --- | --- | --- |
| **Argentina** | 24.5 | 9.7 | 41.6 | 62.7 |
| **Bolivia** | 12.7 | 4.4 | (missing) | 56.1 |
| **Brazil** | 12.8 | 7.4 | 47.0 | 56.5 |
| **Chile** | 29.2 | 9.1 | 26.6 | 63.1 |
| **Colombia** | 8.5 | 5.7 | 44.0 | 59 |
| **Ecuador** | 11.3 | 4.2 | 27.2 | 56 |
| **Paraguay** | 11.5 | 7.6 | 37.4 | 53.5 |
| **Peru** | 8.1 | 6.4 | (missing) | 57.5 |
| **Uruguay** | 21.5 | 6.9 | 22.4 | 62.9 |
| **Venezuela** | (missing) | 4.1 | 31.4 | 63.4 |
| **World** | **22.3** | **6.2** | **27.5** | **39** |

***Notes:*** Data are from the World Health Organization [12–15]. **i.** Percentage of the population ages 15 years and older who currently use any tobacco product on a daily or nondaily basis. Data refer to the year 2020. **ii.** Total per capita consumption of pure alcohol in the period 2016–2018. **iii.** Data refer to the year 2016. **iv.** Data refer to the year 2016. A person is defined as overweight if they have a body-mass index that is equal to or larger than 25.

**Table A10. Assumptions about economic parameters used in the macroeconomic model.**

| **Country** | **Annual economic growth rate (%)^i^** | | **Annual saving rate (%)^ii^** | | **Elasticity of output with respect to physical capital^iii^** | **Annual growth rate of proportion of GDP devoted to health spending (%)^iv^** |
| --- | --- | --- | --- | --- | --- | --- |
|  | **Mean** | **Standard deviation** | **Mean** | **Standard deviation** |  |  |
| **Argentina** | 1.84 | 5.60 | 15.85 | 2.75 | 0.45 | 1.02 |
| **Bolivia** | 2.84 | 2.97 | 18.65 | 5.25 | 0.50 | 1.02 |
| **Brazil** | 2.25 | 3.19 | 15.35 | 2.38 | 0.42 | 1.00 |
| **Chile** | 3.93 | 4.31 | 21.78 | 1.96 | 0.56 | 1.02 |
| **Colombia** | 3.45 | 2.71 | 17.03 | 1.86 | 0.50 | 1.02 |
| **Ecuador** | 2.69 | 3.28 | 26.00 | 1.18 | 0.33 | 1.03 |
| **Paraguay** | 3.31 | 3.24 | 23.15 | 1.31 | 0.55 | 1.02 |
| **Peru** | 3.21 | 5.50 | 21.23 | 1.27 | 0.55 | 1.01 |
| **Uruguay** | 2.24 | 4.07 | 15.51 | 1.80 | 0.53 | 1.00 |
| **Venezuela** | -0.84 | 9.99 | 14.85 | 9.22 | 0.57 | 0.97 |

***Notes:*** **i.** Annual economic growth rate past year 2027 (2023 for Venezuela) equals the average GDP growth rate over the period 1980–2027 (1980–2023 for Venezuela). **ii.** Annual saving rate equals the average value over the period 2010–2021. For Venezuela, data are available only for the period 2010–2014, and we assume that the 2014 saving rate applies to all years from 2015 to 2021. Data on gross saving rate (as % of GDP) are from the World Bank [16]. **iii.** Penn World Table v.10, 2019 data [17]. **iv.** The annual growth rate of health expenditures equals the average value over the period 2000–2019.

**B. Description of the Macroeconomic Model**

GDP in period $t$ is modeled as a function of effective labor supply $H_{t}$, physical capital stock $K_{t}$, and technological progress $A_{t}$:

$$Y_{t}=A_{t}H_{t}^{1-\alpha}K_{t}^{\alpha}$$

with $\alpha$ as the elasticity of final output with respect to capital. Effective labor supply$H_{t}$ accounts for the number of individuals participating in the labor force and for their level of human capital [18]. The model accounts for age differences in labor force participation and human capital levels:

$$H_{t}=\sum_{a} h_{t}^{a}{l_{t}^{a}N}_{t}^{a}$$

where $h_{t}^{a}$ is average human capital at age $a$, $l_{t}^{a}$ is labor force participation at age $a$, and $N_{t}^{a}$ is the number of age-$a$ individuals. Following the Mincer model [19], human capital depends on individuals’ educational attainment and their work experience:

$$\ln h_{t}^{a}=\eta_{1}ys_{t}^{a}+\eta_{2}\left( a-ys_{t}^{a}-5 \right)+\eta_{3}\left( a-ys_{t}^{a}-5 \right)^{2}$$

where $\eta_{1}$ is the semi-elasticity of human capital with respect to average years of education $ys_{t}^{a}$ of the age cohort $a$, and $\eta_{2}$ and $\eta_{3}$ are the semi-elasticities of human capital with respect to experience of the workforce $\left( a-ys_{t}^{a}-5 \right)$ and experience of the workforce squared, respectively. Work experience is proxied by the potential number of years in the workforce, accounting for the length of studies and the age at which children enter school (here assumed to be 5).

Health enters the model in three ways. First, noncommunicable diseases and mental health conditions (NMHs) affect mortality patterns over time and therefore the number of working-age individuals ($N_{t}^{a}$). Second, NMHs cause disabilities that further affect the labor force participation of individuals ($l_{t}^{a}$). Thus, reductions in NMH prevalence positively affect the size of the labor force and thus increase GDP. Third, NMHs negatively affect physical capital accumulation because savings finance part of the treatment costs:

$$K_{t+1}=\left( 1-\delta\right)K_{t}+Y_{t}-C_{t}-\chi TC_{t}=\left( 1-\delta\right)K_{t}+s_{t}Y_{t}$$

where $\delta$ is the depreciation rate of physical capital $K_{t}$, $C_{t}$ is aggregate consumption, $TC_{t}$ denotes the treatment costs associated with NMHs, $\chi\in[0,1]$ is the proportion of treatment costs financed through savings, and $s_{t}$ is the saving rate. Reduction of NMH prevalence saves healthcare resources that can be invested in physical capital.

From the previous equation, it follows that the saving rate is defined as

$$s_{t}=1-\frac{C_{t}+TC_{t}}{Y_{t}}$$

To determine the overall macroeconomic burden of NMHs, we simulate and compare GDP for each country over three decades (2020–2050) in two scenarios. In the *status quo scenario*, we assume that prevalence of NMHs and GDP evolve as expected from recent trends. In the *counterfactual scenario*, we assume complete elimination of NMHs at zero cost. The economic burden of NMHs is given by the discounted cumulative difference in GDP across the two scenarios.

Following Bloom et al. [20], the evolution of labor supply in the status quo scenario is given by $L_{t}^{a}=l_{t}^{a}N_{t}^{a}$, with

$N_{t}^{a}=\left[ 1-\sigma_{t-1}^{a-1} \right]N_{t-1}^{a-1}$,

where $\sigma_{t}^{a}$ is the overall mortality rate of age group $a$ at time $t$. Denote the mortality rate of people in age group $a$ due to NMHs by $\sigma_{d,t}^{a}$, and let $\sigma_{-d,t}^{a}$ be the overall mortality rate caused by other diseases. Then, we have

$$\left( 1-\sigma_{t}^{a} \right)=\left( 1-\sigma_{dt}^{a} \right)(1-\sigma_{-dt}^{a})$$

In the counterfactual scenario, NMHs are fully eliminated, and the size of the cohort aged $a$ at time $t$, $\bar{N_{t}^{a}}$, evolves according to

$$\bar{N_{t}^{a}}=\left[ 1-\sigma_{-d,t-1}^{a-1} \right]\bar{N_{t-1}^{a-1}}$$

with $\bar{N_{0}^{a}}=N_{0}^{a}$ and $\bar{N_{t}^{0}}=N_{t}^{0}$. Following Bloom et al. [20], the loss of labor due to mortality accumulates over time according to

$$\bar{N_{t}^{a}}=\frac{N_{a}^{a}}{\prod_{\tau=0}^{\min\left\{ t,a \right\}-1} \left[ 1-\sigma_{d,t-1-\tau}^{a-1-\tau} \right]}$$

The morbidity effect is captured through reduction in the labor force participation rate $l_{t}^{a}$. Following Bloom et al. [20], the labor force participation rate in the counterfactual scenario can be calculated as

$$\bar{l_{t}^{a}}\boldsymbol{\simeq}\frac{l_{t}^{a}}{\prod_{\tau=0}^{\min\left\{ t,a \right\}-1} \left[ 1-p^{\tau}\sigma_{d,t-1-\tau}^{a-1-\tau}\xi^{a-1-\tau} \right]}$$

where $\xi^{a}$ measures the size of the morbidity effect relative to the mortality rate, and $p^{\tau}$ is the probability that a person affected by NMHs fails to recover until time $\tau$.

Because of lack of data on the impact of morbidity on labor supply, we define

$$\xi^{a}=\frac{loss of labor due to morbidity in age group a}{loss of labor due to mortality in age group a}$$

and we assume that the following relation holds:

$$\xi^{a}=\frac{YLD^{a}}{YLL^{a}}$$

where $YLD^{a}$ represents the years of life lived with a disease and $YLL^{a}$ represents the years of life lost due to the disease. The variable $\xi^{a}$ can be computed using data from the 2019 Global Burden of Disease Study [1].

The overall change in labor supply can be approximated as

$$\Delta L_{t}^{a}\simeq l_{t}^{a}N_{t}^{a}\sum_{\tau=0}^{\min\left\{ t,a \right\}-1} \sigma_{d,t-1-\tau}^{a-1-\tau}\left[ 1+p^{\tau}\xi^{a-1-\tau} \right]$$

Physical capital accumulation in the counterfactual scenario can be written as

$$\bar{K}_{t+1}=\bar{s}_{t}\bar{Y}_{t}+\left( 1-\delta\right)\bar{K}_{t}$$

$$\bar{s}_{t}\bar{Y}_{t}=s_{t}\bar{Y}_{t}+\chi TC_{t}$$

The counterfactual saving rate is thus defined as

$$\bar{s}_{t}=\frac{s_{t}\bar{Y}_{t}+\chi TC_{t}}{\bar{Y}_{t}}$$

To solve the model, we first use projections of GDP, effective labor supply, and physical capital stock in the status quo scenario to determine the technological progress variable $A_{t}$. Then, we simulate trajectories of effective labor supply and physical capital stock in the counterfactual scenarios, and, together with the estimated values of technological progress $A_{t}$, we compute the counterfactual GDP trajectories.

**C. Calibration of the Macroeconomic Model**

This section describes the calibration of the model and methodology used to project future health and economic variables in the status quo scenario.

***Population***

Age-specific population projections from 2020 to 2050 are from the 2022 World Population Prospects [4]. In the simulations we consider the following age groups: 0–4, 5–9, 10–14, 15–19, 20–24, 25–29, 30–34, 35–39, 40–44, 45–49, 50–54, 55–59, 60–64, 65–69, and 70+. We also distinguish projections by gender.

***Mortality Rates***

Disease-, gender-, and age-specific mortality rates are from the 2019 Global Burden of Diseases Study (GBD) [1]. To project future mortality rates, we assume that mortality rates grow at a constant yearly disease-, gender-, and age-specific rate. We assume that such a rate is normally distributed, and we estimate the mean and standard deviation of its distribution using GBD mortality rates from the period 2010–2019. In the baseline scenarios, we rely on the mean GBD mortality estimates, and in the sensitivity analyses we include results for the 2.5 (“low health burden”) and 97.5 (“high health burden”) percentile estimates provided by GBD.

***Morbidity Rates***

Absent country-specific data on the impact of having a disability on labor force participation and productivity, the model assumes that mortality and morbidity contribute to reduction in labor supply in the same proportions that mortality and morbidity contribute to the health burden of NMHs in terms of DALYs, where mortality is measured in terms of years of life lost (YLLs) and morbidity in terms of years of life lived with a disease/disability (YLDs) [20]. More specifically, the reduction in labor force participation rate due to morbidity is estimated to equal $\frac{YLD_{at}}{YLL_{at}}*mortality rate_{at}$. Data on DALYs, YLLs, and YLDs are from GBD. Projections about future YLDs and YLLs assume a constant yearly growth rate based on data over the period 2010–2019, as in the projections for future mortality rates.

***Labor Force Participation Rates***

Labor force participation rates are from the International Labour Organization (ILO), which provides gender-specific data in five-year age bands up to 2021. We use annual ILO-modeled estimates data about labor force participation rate by sex and age [21]. To project future labor force participation rates, we assume a constant age- and gender-specific annual growth rate. We assume that the annual growth rates are normally distributed and derive the mean and standard deviations based on labor force participation rates from the period 2010–2021.

***Education***

We rely on the Barro-Lee database for country-, gender-, and age-specific data on average years of schooling [22]. The database provides data up to 2015 (September 2021 update) and country- and gender-specific projections of educational attainment for the populations aged 15–24 and 25–64 up to 2040. The Barro-Lee database provides data in five-year intervals; linear interpolation was adopted to determine annual estimates. Past year 2040, we assume a constant increase in years of schooling equal to the age- and gender-specific rate estimated in 2040. The parameters of the Mincer equation are from Heckman et al. (2006): $\eta_{1}=0.091$, $\eta_{2}=0.1301$, and $\eta_{3}=-0.0023$ [23].

***Gross Domestic Product***

For each country, GDP projections in the status quo scenario are derived by combining data on purchasing power parity (PPP) GDP from World Bank [2] (in 2017 international $, most recent year is 2021) and projected real GDP growth rates from the International Monetary Fund (IMF) [24]. For Venezuela, we rely on PPP GDP data from the Central Intelligence Agency’s Factbook (most recent year is 2018) [6]. IMF’s projected growth rates are available until 2027 (2023 for Venezuela). To project GDP growth rates up to 2050, we assume that the annual economic growth rate in the period 2028–2050 (2024–2050 for Venezuela) is normally distributed, and we use economic growth rates observed since 1980 to estimate the mean and the standard deviation of the distribution. Table S10 summarizes the results.

***Savings***

Data on the stock of physical capital at the beginning of the simulation are from the Penn World Table (the latest available values are from 2019) [17]. The calibration of the elasticity of output with respect to physical capital is also based on data from the Penn World Table, with elasticity equal to one share of labor compensation in GDP [25]. In the status quo scenario, annual saving rates are kept constant and assumed to be normally distributed, with mean and standard deviations based on saving rates in the years 2010–2021. Data on gross savings (as % of GDP) are from the World Bank [16]. Table S10 summarizes the results.

***Treatment Costs***

To estimate country-level treatment costs, we assume that the per case treatment cost for a specific disease is proportional to the per capita health expenditure in a country, as previous studies have assumed [26–28]:

$$Per case treatment cost_{d}=\beta_{d}*Per capita health expenditures$$

where $\beta_{d}$ denotes the proportion of per capita health expenditures devoted to paying treatment costs for one case of disease $d$. Total treatment costs for a specific disease equal

$$Treatment costs_{d}=\beta_{d}*prevalence_{d}*per capita health expenditures*population$$

Dieleman et al. [29] provide estimates of the fraction of total health expenditures devoted to a particular disease, i.e., the term $\beta_{d}*prevalence_{d}$. Assuming that the proportion $\beta_{d}$ is constant in all countries, we then derive country- and disease-specific per capita treatment costs by multiplying the estimated proportion by country-specific data on disease prevalence and per capita health expenditures. To account for rising medical costs, we assume that treatment costs will increase over time. We project future health expenditures using World Bank data on health expenditures (as % of GDP) for the years 2000–2019 [5] and assuming that in the future they will grow at a constant yearly rate. Table S10 provides the health expenditure growth rates. Table S2 provides data on health expenditures (as % of GDP).

**D. Conversion to 2022 International $**

All initial results were expressed in 2017 international $, and we converted them into 2022 international $ using a conversion rate of 1.19. The conversion rate was computed using a geometric average of monthly U.S. Consumer Price Index inflation rates from 2017 to 2022 as published by the Bureau of Labor Statistics. Data on inflation rates have been retrieved from <https://data.bls.gov/cgi-bin/cpicalc.pl?cost1=1.00&year1=201701&year2=202201>.

**E. Alternative Evaluation Methods**

We compare the economic burden estimates of NMHs in South America with the results derived from two alternative economic evaluation methodologies: the cost-of-illness (COI) approach and the value-per-statistical-life (VSL) approach.

***Cost-of-illness approach***

In the COI approach, the cost of a health condition is given by the sum of its direct and indirect costs. Due to data limitations, we consider two sets of costs: treatment costs (direct costs) and income losses (indirect costs). We assume that only the working population suffers an income loss due to NMHs, and we proxy the income loss by GDP per capita, $pcGDP_{t}$. All direct and indirect future costs are discounted at a constant yearly rate $r$. Thus, the cost of illness associated with NMHs in a given country is given by

$$COI=\sum_{t=2020}^{2050} \frac{1}{\left( 1+r \right)^{t-2020}}\left( pcGDP_{t}*\sum_{a=15}^{64} {\Delta L}_{at}+TC_{t}^{NMHs} \right)$$

where ${\Delta L}_{at}$ is the change in labor force of individuals at age $a$, and $TC_{t}^{NMHs}$ are the treatment costs due to NMHs paid in year $t$. We assume that only individuals in the 15-64 age-range participate in the labor force. For both treatment costs and changes in labor force, we use the projections derived in the calibration of the macroeconomic model.

***Value-per-statistical-life approach***

The VSL approach captures both the intrinsic and instrumental value of being alive and in good health. We assume that both the value of an additional year of life and the value of a year of life lived without a disability are equal to a constant time-specific number, the value-per-statistical-life-year (VSLY). Following the relevant literature, we assume that the VSLY is 1 to 5 times GDP per capita, and we discount future economic costs at the constant yearly rate $r$. The economic burden of NMHs is given by

$$\sum_{t=2020}^{2050} \frac{1}{\left( 1+r \right)^{t-2020}}VSLY_{t}*DALY_{t}$$

where $VSLY_{t}=\alpha*pcGDP_{t}$, $\alpha$ is a constant that takes a value from 1 to 5, and $DALY_{t}$ is the total number of DALYs lost due to NMHs in year $t$. To derive the number of DALYs lost, we first project the number of DALYs associated with NMHs by country, gender, and age group. As in the projection of mortality and morbidity rates in the macroeconomic model, we use disease-, gender-, and age-specific DALY rates (number of DALYs per 100,000 people) from GBD. To project future DALY rates, we assume that DALY rates grow at a constant yearly disease-, gender-, and age-specific rate. We assume that such a rate is normally distributed, and we estimate the mean and standard deviation of its distribution using mean estimates of GBD DALY rates from the period 2010–2019. We then multiply the projected DALY rates by future population sizes to determine the number of DALYs by year, country, gender, and age group.

**References**

1. Vos T, Lim SS, Abbafati C, Abbas KM, Abbasi M, Abbasifard M, et al. Global Burden of 369 Diseases and Injuries in 204 Countries and Territories, 1990–2019: A Systematic Analysis for the Global Burden of Disease Study 2019. The Lancet. 2020; 396: 1204–1222. doi:10.1016/S0140-6736(20)30925-9

2. World Bank. GDP PPP, constant 2017 international $. [cited 3 Aug 2023]. Available: https://data.worldbank.org/indicator/NY.GDP.MKTP.PP.KD

3. World Bank. GDP per capita, PPP. [cited 3 Aug 2023]. Available: https://data.worldbank.org/indicator/NY.GDP.PCAP.PP.CD

4. United Nations Population Division. World Population Prospects 2022. 2022. [cited 8 Feb 2023] Available: https://www.un.org/development/desa/pd/sites/www.un.org.development.desa.pd/files/wpp2022_summary_of_results.pdf

5. World Bank. Current health expenditures (% of GDP). [cited 3 Jun 2023]. Available: https://data.worldbank.org/indicator/SH.XPD.CHEX.GD.ZS

6. Central Intelligence Agency. The World Factbook, 2021. Washington, DC; 2021.

7. World Bank. Government expenditures on education, total (% GDP) . [cited 3 Aug 2023]. Available: https://data.worldbank.org/indicator/SE.XPD.TOTL.GD.ZS

8. World Bank. Tax revenue (% GDP). [cited 3 Aug 2023]. Available: https://data.worldbank.org/indicator/GC.TAX.TOTL.GD.ZS

9. World Bank. Military expenditure (% GDP). [cited 3 Aug 2023]. Available: https://data.worldbank.org/indicator/MS.MIL.XPND.GD.ZS

10. World Bank. Public and publicly guaranteed debt service (% of GNI). [cited 3 Aug 2023]. Available: https://data.worldbank.org/indicator/DT.TDS.DPPG.GN.ZS

11. World Bank. GNI, PPP (constant 2017 international $). [cited 3 Aug 2023]. Available: https://data.worldbank.org/indicator/NY.GNP.MKTP.PP.KD

12. World Health Organization. Prevalence of overweight among adults, BMI >=25 (age-standardized estimate) (%). The Global Health Observatory. Geneva: World Health Organization; 2022.

13. World Health Organization. Prevalence of insufficient physical activity among adults aged 18+ years (age-standardized estimate) (%). The Global Health Observatory. Geneva: World Health Organization; 2022.

14. World Health Organization. Prevalence of current tobacco use among persons aged 15 years and older (age-standardized rate). The Global Health Observatory. Geneva: World Health Organization; 2022.

15. World Health Organization. Alcohol, total per capita (15+ years) consumption (in litres of pure alcohol). The Global Health Observatory. Geneva: World Health Organization; 2022.

16. World Bank. Gross savings (% of GDP). [cited 3 Aug 2023]. Available: https://data.worldbank.org/indicator/NY.GNS.ICTR.ZS

17. Feenstra RC, Inklaar R, Timmer MP. The Next Generation of the Penn World Table. American Economic Review. 2015; 105: 3150–3182. doi:10.1257/aer.20130954

18. Lucas RE. On the Mechanics of Economic Development. J Monet Econ. 1988; 22: 3–42. doi:10.1016/0304-3932(88)90168-7

19. Mincer J. Schooling, Experience, and Earnings. New York: National Bureau of Economic Research; distributed by Columbia University Press; 1974.

20. Bloom DE, Chen S, Kuhn M, McGovern ME, Oxley L, Prettner K. The Economic Burden of Chronic Diseases: Estimates and Projections for China, Japan, and South Korea. The Journal of the Economics of Ageing. 2020; 17: 100163. doi:10.1016/j.jeoa.2018.09.002

21. International Labour Organization. Labour force participation rate by sex and age—ILO modelled estimates, Nov. 2022 (%) – annual. In: ILOSTAT [Internet]. Geneva; 2022 [cited 3 Aug 2023]. Available: https://www.ilo.org/shinyapps/bulkexplorer36/?lang=en&segment=indicator&id=EAP_TEAP_SEX_AGE_NB_A

22. Barro RJ, Lee J-W. Education Matters. Oxford University Press; 2015. doi:10.1093/acprof:oso/9780199379231.001.0001

23. Heckman JJ, Lochner LJ, Todd PE. Chapter 7 Earnings Functions, Rates of Return and Treatment Effects: The Mincer Equation and Beyond. Handbook of the Economics of Education. 2006; 1: 307–458. doi:10.1016/S1574-0692(06)01007-5

24. International Monetary Fund. World Economic Outlook 2022. Washington DC: International Monetary Fund; 2022.

25. Hall RE. The Relation Between Price and Marginal Cost in U.S. Industry. Journal of Political Economy. 1988; 96: 921–947. doi:10.1086/261570

26. Bloom DE, Cafiero ET, Jané-Llopis E, Abrahams-Gessel S, Bloom LR, Fathima S, et al. The Global Economic Burden of Noncommunicable Diseases. Geneva: World Economic Forum; 2011.

27. Chen S, Kuhn M, Prettner K, Bloom DE. Noncommunicable Diseases Attributable to Tobacco Use in China: Macroeconomic Burden and Tobacco Control Policies. Health Aff. 2019; 38: 1832–1839. doi:10.1377/hlthaff.2019.00291

28. Ding D, Lawson KD, Kolbe-Alexander TL, Finkelstein EA, Katzmarzyk PT, van Mechelen W, et al. The Economic Burden of Physical Inactivity: A Global Analysis of Major Non-Communicable Diseases. The Lancet. 2016; 388: 1311–1324. doi:10.1016/S0140-6736(16)30383-X

29. Dieleman JL, Cao J, Chapin A, Chen C, Li Z, Liu A, et al. US Health Care Spending by Payer and Health Condition, 1996–2016. JAMA. 2020; 323: 863. doi:10.1001/jama.2020.0734
